# Chemiluminescence Detection in Flowing Streams—Immobilized and Solid-State Reagents

**DOI:** 10.6028/jres.093.135

**Published:** 1988-06-01

**Authors:** Timothy A. Nieman

**Affiliations:** Department of Chemistry, University of Illinois, 1209 W. California St. Urbana, IL 61801

Chemiluminescence (CL) reactions yield light as one of their products. Luminol (5-amino-2,3-dihydro-1,4-phthalazinedione) is one of the CL reagents most commonly used in analysis. In aqueous alkaline solution, luminol is oxidized to yield 3-aminophthalate and light. Perhaps the most analytically useful oxidant is hydrogen peroxide, for which a reaction catalyst is required. Typical catalysts are peroxidase, hemin, transition metal ions, or ferricyanide. The luminol CL system most often used consists of luminol + H_2_O_2_ + catalyst + OH^−^. Measurements of CL intensity can be used to quantitate any of these species. Thus, luminol CL has been used to determine catalysts or species labelled with catalysts, peroxide, or species which can be converted into peroxide, and luminol, or species labelled with luminol.

For quantitative determination of any one species involved in this reaction, one needs a way to deliver the other reaction components into the reaction zone or vessel. In flowing stream situations, either flow injection analysis or liquid chromatography, one would have a stream of analyte into which were flowed streams of the other necessary reagents as shown in figure 1. If the analyte were peroxide, then reagents 1 and 2 would be luminol and catalyst.

Such a system requires solutions of these reagents plus the necessary pumps, tubing, and mixers to deliver them and results in dilution and broadening of the sample plug. Our research effort has explored various ways to contain these reagent components in solid-state or immobilized format so that the usual reagents solutions, pumping, and tubing can be eliminated, and the reagents incorporated in-line as indicated in figure 2.

Immobilized luminol is of use for determinations of catalyst species or for determination of peroxide (and species such as glucose, cholesterol, etc., which can be enzymatically converted into an equivalent amount of peroxide). We have covalently bound luminol to silica, controlled-pore glass, and Ambersorb particles via various silane and linkage molecules. By using glutaraldehyde to bridge between the amine group on luminol and the amine group on an aminoalkylsilane, loadings of 15 mg luminol/g support are achieved for supports with about 400 square meters surface area/gram. This material is packed into a column placed into the flow stream. The immobilized luminol is stable in neutral solution, but injection of a portion of an alkaline solution will release a controlled amount of luminol (via hydrolysis of the bond between luminol and glutaraldehyde); the amount released is dictated by the pH and volume of the solution injected. We have also contained luminol simply by adsorption onto Ambersorb and released it in the same manner. Advantages to release of luminol prior to the CL reaction include improvement in the CL quantum efficiency and increased flexibility in engineering the contact with the other reagents. By use of this material in flow injection, a detection limit of 0.1 μM for peroxide is obtained.

An immobilized catalyst is of use for determination of peroxide (and analytes enzymatically converted to peroxide) or analytes that have been labeled with luminol or related compounds. An approach that we have investigated involves use of electrogeneration of luminol CL. Luminol can be oxidized at a positively-biased electrode, and CL results if oxygen or hydrogen peroxide are present. Electrogeneration of luminol CL offers the unique characteristics that the CL emission is confined to near the electrode surface and that the reaction can be turned “on” and “off” via control of electrode potential. The net electrogenerated CL (ECL) intensity due to peroxide injections appears as peaks on top of an appreciable and inescapable background due to oxygen. This background ECL however, does not prevent one from obtaining peroxide detection limits below 1 μM. The ECL intensity varies with both pH and potential. The net ECL to background ECL ratio is a maximum at pH 10. Gold and glassy carbon electrodes are preferable to platinum, but gold is somewhat better than glassy carbon. Potentials more negative than + 0.3 V yield insignificant ECL; potentials of +0.4 to +0.6 V yield maximum ECL intensity. Long-term stability of the ECL signal is improved by use of a potential waveform which alternates between a positive potential where luminol is oxidized and a negative potential (about −0.2 V) where the electrode surface is reduced.

If luminol or luminol-labeled species are the analyte, then one needs both peroxide and catalyst as the reagents. Because the catalyst can be achieved electrochemically, it makes sense also to consider electrochemical generation of the peroxide from oxygen and water. In a stream flowing through an electrochemical flow cell, as long as the oxygen concentration, pH, flow rate, and electrode behavior remain constant, the amount of peroxide produced at the electrode per unit time will remain constant so that a constant concentration of peroxide will be achieved in the flowing stream. Use of a glassy carbon generating electrode, poised at about −1.0 V, yields sufficiently high peroxide concentrations over the pH 9.5 to 11 range to be useful with luminol CL; those solution pH values are also compatible with the luminol CL reaction. By using two electrodes in series (an upstream electrode to generate peroxide and a downstream electrode for luminol ECL) we have determined luminol from 0.1 nM to 10 μM.

## Figures and Tables

**Figure 1:**
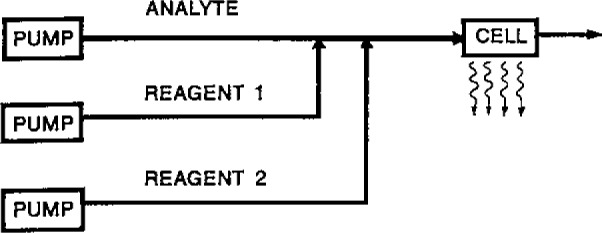
Typical chemiluminescence flow system using dissolved reagents.

**Figure 2:**
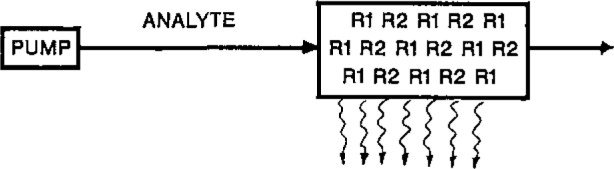
Possible chemiluminescence flow system with immobilized reagents (R1 and R2).

